# Glycation Damage: A Possible Hub for Major Pathophysiological Disorders and Aging

**DOI:** 10.14336/AD.2017.1121

**Published:** 2018-10-01

**Authors:** Maxime Fournet, Frédéric Bonté, Alexis Desmoulière

**Affiliations:** ^1^University of Limoges, Faculty of Pharmacy, Department of Physiology, EA 6309, F-87025 Limoges, France; ^2^LVMH Recherche, F-45800 St-Jean-de-Braye, France; ^3^University of Limoges, Faculty of Pharmacy, Department of Physiology, EA 6309, F-87025 Limoges, France

**Keywords:** exogenous glycation, endogenous glycation, advanced glycation end product, aging, neurodegenerative disorders, diabetes

## Abstract

Glycation is both a physiological and pathological process which mainly affects proteins, nucleic acids and lipids. Exogenous and endogenous glycation produces deleterious reactions that take place principally in the extracellular matrix environment or within the cell cytosol and organelles. Advanced glycation end product (AGE) formation begins by the non-enzymatic glycation of free amino groups by sugars and aldehydes which leads to a succession of rearrangements of intermediate compounds and ultimately to irreversibly bound products known as AGEs. Epigenetic factors, oxidative stress, UV and nutrition are important causes of the accumulation of chemically and structurally different AGEs with various biological reactivities. Cross-linked proteins, deriving from the glycation process, present both an altered structure and function. Nucleotides and lipids are particularly vulnerable targets which can in turn favor DNA mutation or a decrease in cell membrane integrity and associated biological pathways respectively. In mitochondria, the consequences of glycation can alter bioenergy production. Under physiological conditions, anti-glycation defenses are sufficient, with proteasomes preventing accumulation of glycated proteins, while lipid turnover clears glycated products and nucleotide excision repair removes glycated nucleotides. If this does not occur, glycation damage accumulates, and pathologies may develop. Glycation-induced biological products are known to be mainly associated with aging, neurodegenerative disorders, diabetes and its complications, atherosclerosis, renal failure, immunological changes, retinopathy, skin photoaging, osteoporosis, and progression of some tumors.

The French chemist, Louis-Camille Maillard, published an article in 1912 describing the changes that occur in specific proteins once they attach to a sugar. This series of reactions known as the "Maillard reaction" is the main reason why food develops color (browning) and aromas when it is cooked (exogenous glycation). Maillard, a pioneering scientist, speculated that this reaction could play a role in a variety of fields, including physiology, human pathology, and agronomy. Other scientists subsequently studied these reactions including Amadori, at the end of the 1920s, and Hodge in the early 1950s. Glycated hemoglobin, discovered in 1955, was the first example of a molecule that had been altered by exposure to blood sugar and provided proof that the Maillard reaction also occurred in the human body (endogenous glycation). Biologists now use the term glycation to describe the Maillard reaction *in vivo*.

Glycation is one of the fundamental mechanisms involved in aging and is known to occur at different reactive rates in young people compared to older people. Moreover, glycation is also recognized to be more associated with diabetes mellitus. While glycation products occur in healthy subjects and contribute to the aging process, long-term hyperglycemia, in diabetic subjects, results in an increase in these products, triggering toxic mechanisms that have a more serious impact on health. In addition, nutrition during early development plays an important role in susceptibility to disease in adult life. This suggests potential links between the nutrition status or diet and changes in gene expression that may lead to a predisposition to disease phenotypes [1]. The consumption of glycation products, especially those formed during cooking, could also be potentially harmful to our health.

**Figure 1.**
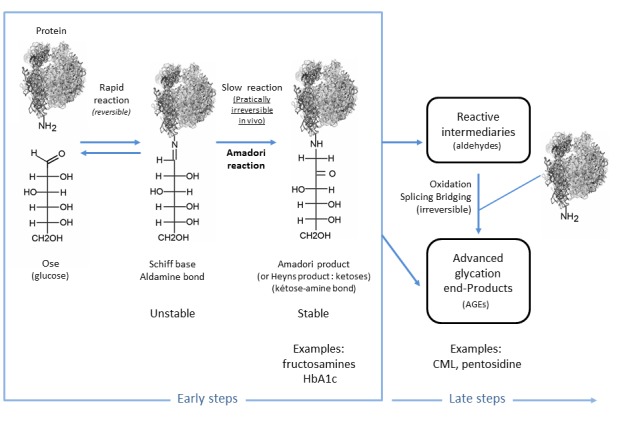
*In vivo* glycation processes.

## General mechanisms of exogenous and endogenous glycation

Glycation is a non-enzymatic reaction that occurs between a carbohydrate and a molecule with a free amino group, such as a protein (Fig. 1). This irreversible and cumulative reaction takes place spontaneously in the body. All resulting metabolic intermediates are then reactive. Glycation is a physiological and pathological process that produces so-called glycated proteins and is entirely separate from glycosylation, which represents an enzyme-controlled physiological process that occurs during synthesis of glycoproteins. When it occurs outside of the body, glycation is responsible for the browning process that occurs in food during cooking [2]. Exogenous glycation is the result of a covalent bond between a carbonyl group from a reducing sugar (aldose or ketose) and a free amino group. Through a reversible reaction, exogenous glycation forms a Schiff base after the liberation of a water molecule. After the Schiff base is irreversibly isomerised, Amadori products are formed from aldoses and Heyns-Carson products are formed from ketoses. Once these products fragment, in a process known as the retro-aldol reaction, they create a series of aldehydes and carbonyl compounds including glyceraldehyde, formaldehyde, and glyoxal, which in turn can react with another amino group. The compounds formed by splitting these products include important intermediary molecules that are also created during endogenous glycation; these are known as glycation intermediaries. Glycation intermediaries can continue to react, creating advanced glycation end products, or AGEs (Fig. 1). AGEs refer to the end products formed by endogenous glycation. An AGE, such as N-ε-carboxymethyl-lysine (CML) or N-ε-carboxyethyl-lysine (CEL), can also form during exogenous glycation and be present in food [3].

Endogenous physiological glycation involves glucose, the body’s most prevalent reducing sugar, and the functions of free amino groups present in the body as well, especially amino acids in proteins like lysine and arginine. It has also been shown to play a role in numerous pathologies in diabetic and non-diabetic patients alike. Glycation increases in patients with type I diabetes due to pancreatic beta cell failure and the consequent loss of control of blood glucose which leads to hyperglycemia. In type II diabetes, insulin resistance similarly results in hyperglycemia. The body’s proteins thus come into contact with high levels of blood sugar (chronic hyperglycemia). As a result, glycation is accelerated and contributes to the various complications associated with diabetes [4-6]. The resulting carbonyl molecules tend to accumulate in the bodies of subjects suffering from diabetes or from kidney failure [7-10].

These intermediaries are very reactive and cause carbonyl stress, which in turn can aggravate inflammation and oxidative stress.

**Figure 2.**
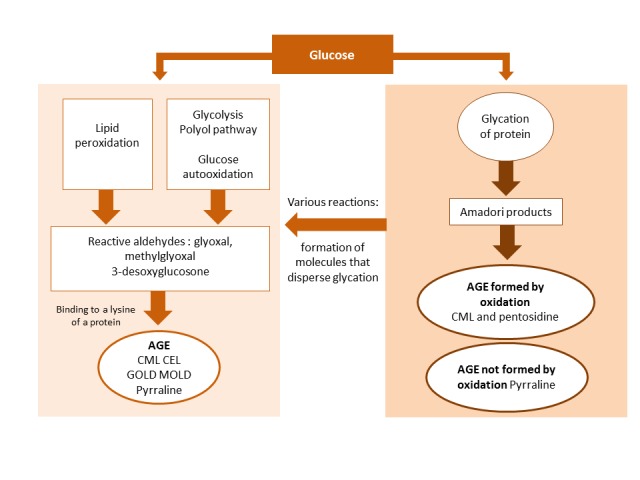
All AGEs formed in the body due to glycation and four other metabolic pathways.

The reactive intermediaries described above are located at the "crossroads" of various metabolic pathways. The polyol, glycolysis, glucose autoxidation, and lipid peroxidation pathways create these same glycation intermediaries (Fig. 2).

Once formed, these intermediaries react, just as they would during the glycation process, with amino acids in proteins such as lysine, to produce AGEs (CML, CEL, or pyrraline) without previously undergoing glycation. It is for this reason that the term AGE strictly refers to all AGEs produced through glycation, plus the advanced end products formed by the pathways mentioned above. This is also why AGEs have a variable "pathological expression" in diabetes mellitus, kidney failure, and tissue aging. Aminophospholipids, which are found in cellular and mitochondrial membranes, phosphatidyl-ethanolamines, and serines are present *in vivo* in a glycated form. The resulting structural changes affect phase transitions, which in turn alters membrane plasticity, membrane potential, and conductance. The pro-oxidative role of the glycated forms is also associated with cellular responses such as the activation of the transcription factor nuclear factor-κB (NF-κB). They also have a significant impact on mitochondria since they disrupt the regulation of autophagy and cellular bioenergy production [11-15].

Interestingly, Takeuchi et al, [16] have provided direct immunochemical evidence for the existence of six distinct AGE structures (AGE-1, glucose-derived AGEs; AGE-2, glyceraldehyde-derived AGEs; AGE-3, glycolaldehyde-derived AGEs; AGE-4, methylglyoxal-derived AGEs; AGE-5, glyoxal-derived AGEs; and AGE-6, 3-deoxyglucosone-derived AGEs) within the AGE-modified proteins and peptides that circulate in the serum of diabetic patients undergoing hemodialysis. Additionally, among the various types of AGE structures that can form *in vivo*, the AGE-2 structure is likely to play an important role in the pathophysiological processes associated with AGE formation.

## Factors influencing glycation

The glycation reaction is facilitated by low water concentrations as the first step in the reaction is dehydration. However, if the water content is too low, the reagents become less soluble, which inhibits the reaction.

The Maillard reaction is accelerated in alkaline environments due to the increased reactivity of basic free amino groups in proteins. Low pH values result in enolisation 1,2, which creates furfural and its derivatives. The speed of the reaction is strongly influenced by temperature as reaction speed increases exponentially with increased temperature. Temperature also affects the nature of the compounds that are formed. High temperatures lead to the formation of melanoidins. These products cannot be synthesized at 37°C by endogenous glycation [17, 18]. Generally speaking, metals also facilitate this reaction. In addition, Yen and Lai showed in 1987 that the presence of antioxidants such as α-tocopherol and butylhydroxyanisole decreases glycation [19]. Only amino acids located at the protein N-terminus or those with amino acid side chains (lysine, arginine, and histidine) are targeted by glycation.

In the case of short-lived proteins, glycation results in the formation of early glycation products such as Amadori products. This is because of the rapid turnover of these proteins, which means the time is not sufficient for the formation of AGEs, as this slow process takes several weeks or even months. Conversely, the slow turnover of other types of proteins, for example type I collagen, which is abundant in the connective tissues of many organs including skin, means that a complete glycation reaction can occur, resulting in the formation of AGEs [20, 21]. The reactivity of carbohydrates decreases as their size increases. Pentoses are thus more reactive than hexoses. The low reactivity of the carbohydrates most commonly found in humans is due to their extremely stable cyclical structure [22, 23].

## Role played by endogenous glycation products in harmful mechanisms

Glycation affects all of the body’s proteins, including circulating, extracellular, and intracellular proteins. A few examples include hemoglobin, albumin, insulin, immunoglobulins, low-density lipoproteins (LDL), and collagen. Some of the most affected proteins are those that come into direct contact with blood and thus blood glucose, such as albumin, insulin, hemoglobin, immunoglobulins, lipoproteins, and fibrinogen. Other molecules with amino groups, including DNA, are also targeted by glycation [9, 20].

In addition, as previously discussed, long-lived molecules are especially vulnerable to glycation, thus the greatest accumulation of AGEs occurs in these particular proteins. The potentially harmful nature of a glycated protein therefore depends on the duration of its life cycle. While short-lived glycated proteins have little effect, long-lived glycated proteins can lead to the formation of AGEs. These products are not easily eliminated from the body and play a significant role in glucotoxicity. Such is the case for proteins within the extracellular matrix including the various types of collagen and elastin. These molecules have a comparatively slow rate of turnover from a few months to a few years.

Finally, some glycation products form inter-molecular cross-links. Pentosidine is one example of these cross-linking products, which go on to form protein aggregates that accumulate in the body. In addition, some glycation products can trigger inflammatory cells by stimulating the AGE receptor, termed the receptor for advanced glycation end products (RAGE) [9, 21].

## The consequences of glycation-induced structural changes in proteins, lipoproteins, and DNA

### Protein modifications

When a carbohydrate attaches to a protein through the process of fixation, the structural and physical characteristics of that protein are altered. The protein can become resistant to certain enzymes for which it previously acted as the substrate, resulting in an accumulation of that protein. Glycated proteins in the extracellular matrix become less sensitive to proteolysis. For example, glycated collagen fibers within the arterial wall become resistant to remodeling enzymes. As a result, these proteins accumulate, irreversibly thickening the vessel wall. Glycated proteins can also trigger apoptosis in endothelial progenitor cells via NADPH oxidase and promote atherosclerosis [20, 24].

Within tissues, glycation results in protein aggregates due to bonds created by three distinct mechanisms: i) the formation of covalent bonds between glycation end products, ii) the oxidation of sulfur groups (sulfhydryl groups) into disulfide bridges, and iii) the formation of new reactive groups within a protein. Chemical bridges formed by AGEs also result in the reticulation of proteins and their cross-linking (assembly), a phenomenon that occurs within the extracellular matrix and significantly increases the structural rigidity. Glycation can also inhibit the biological effects of some proteins including the effects of hormones (*e.g*. insulin), growth factors, and antibacterial peptides such as lysozyme and lactoferrin [25-30].

### Insulin signaling defect

Insulin is a hormone that lowers blood glucose levels in the body acting through insulin receptors on target cells. Glycation contributes to the cellular mechanisms of insulin resistance. In addition, glycated insulin in pancreatic cells or in the blood makes it harder to maintain homeostatic levels of glucose and to stimulate lipogenesis [20, 27, 28]. Finally, several studies have shown that glycated albumin and methylglyoxal (a glycation intermediary) decrease the biological responses triggered when insulin binds to its receptor on the target cell [29, 30]. Glycation also lowers insulin secretion while AGEs also have a direct effect on the pancreas and create the conditions for oxidation, leading to the malfunction and death of insulin secreting beta cells [28, 31, 32]. All of these factors contribute to the chronic hyperglycemia seen in subjects with type 2 diabetes. This hyperglycemia in turn increases the synthesis of pathological glycated proteins.

### Enzyme activity inhibition

The glycation of enzymes induces conformational changes that may be near the enzyme’s active site [20]. Paraoxonase, a plasma enzyme associated with high-density lipoproteins that prevents the oxidation of LDL and neutralizes oxidized phospholipids that combat the formation of atheromatous plaques, is inhibited by glycation, which can lead to hypertension or potentially thrombosis [33, 34]. In addition, the glycation of enzymes can suppress antioxidant processes in the body. This is due to the glycation of antioxidant enzymes such as copper-zinc superoxide dismutase [35]. In addition, another enzyme, glutathione reductase, produces less intracellular glutathione when glycated, which is normally a powerful antioxidant molecule with detoxifying properties [36, 37].

### Nucleotide changes

Glycated products can react with amino groups in nucleotides. These changes result in breaks to the DNA strand or in mutations that lead to production of abnormal proteins. Numerous epigenetic mechanisms affect the condensation of chromatin and gene accessibility during transcription. Changes to histones and DNA have significant biological consequences [38, 39]. For example, exposure to methylglyoxal or glyoxal, decreased cell growth due to the formation of DNA-protein cross-links and breaks in the cells’ DNA in cultured dermal fibroblasts [35, 40, 41].

### Immune system deficiency

Glycation can also have deleterious effects on normal immune function as immunoglobulins are one of the families of proteins most affected by glycation. Some types of immunoglobulins are more affected than others with the glycation of immunoglobulin M being two times higher than that of immunoglobulin G, due to its different amino acid composition. Glycated immunoglobulin G is less effective in immune function because of an impairment of Fc fragment function, which is involved in complement activation [9, 42]. This process could contribute to immunosuppression in diabetic subjects, rendering them more vulnerable to infections. However, it appears that this type of immune deficiency is more due to a decrease in two sub-types of natural killer cells which normally ingest pathogenic agents and seem to be rendered less effective [43]. Recent studies also suggest there is a link between immune phenomena and the microbiome of diabetic subjects [44, 45].

The glycation of a protein modifies its composition and can also enable it to provoke an immune response. The immune system then produces antibodies against the modified protein. This explains how antibodies targeting AGEs have been observed in both diabetic and healthy subjects [46]. For example, glycated immunoglobulin G antibodies as well as immunoglobulin M autoantibodies that target them appear to be implicated in the pathophysiology of rheumatoid arthritis [47, 48].

In addition, the autoantibodies can bind to circulating AGEs and form immune complexes, which can be found in high concentrations in the blood of diabetic patients [47]. Antibody-glycated-LDL complexes contribute to atherosclerosis, a process that lies at the root of angiopathic complications in diabetes [49, 50].

### Lipoprotein changes

Glycated LDLs are especially abundant in diabetic patients. The glycation of this molecule is often accompanied by oxidation, which contributes to the accumulation of pro-atherogenic oxidized LDL in the blood. Macrophages bind with glycated LDL via their macrophage scavenger receptors and differentiate into foam cells, which then contribute to the formation of plaques in atheroma. In addition, the changes that occur in LDLs during the glycation process give these lipoproteins an immunogenic quality that leads to their accumulation in plasma through the formation of immune complexes. The transport of cholesterol to high-density lipoprotein is also reduced and the beneficial flow of cholesterol from tissues to the liver is therefore likely to be reduced as a consequence [51-54].

## Repair and elimination of glycation products

There are a few hypotheses regarding potential mechanisms that repair glycated proteins. Indeed, there are two intracellular anti-glycation systems that could restore the glycated proteins to their initial form. However, these mechanisms only act on Amadori products and cannot change AGEs. The first of these systems, known as transglycation, separates the carbohydrate portion from the glycated protein and transfers it to an intracellular nucleophile, made up of free amino acids, in a non-enzymatic process. Removing this portion allows the protein to regain its original shape. It is important to note that these nucleophile agents, which include glutathione and carnosine, have an anti-glycation effect. Other molecules, such as pyridoxamine and taurine, combat glycation through different molecular processes. The second repair mechanism uses fructosamine-3-kinase, an enzyme that catalyzes the repair of glycation intermediaries like fructoselysines. The fructosamine-3-kinase enzyme thus limits the formation of glycated hemoglobin in erythrocytes.

However, glycated intracellular proteins are not eliminated by the cell. They remain and may concentrate in and damage cells and tissues. The accumulation of intracellular glycation triggers a series of malfunctions that may end in cellular apoptosis. Extracellular glycated products can also be broken down by proteases. Their elimination through the kidneys is directly dependent on renal function and as a result, in chronic kidney failure, glycated blood components accumulate in the blood and tissues. Indeed, the concentration of plasma AGEs is up to forty times higher in patients on renal dialysis than in healthy patients [55-58]. In addition, as previously discussed, the remodeling enzymes of the extracellular matrix are less effective in degrading glycated proteins; one example of this phenomenon being the reduced effectiveness of matrixins on glycated collagen [21]. This may partially explain the loss of biomechanical properties in soft tissues like the skin.

Glycated products circulating through the bloodstream are removed in the liver by various hepatic cells such as hepatic sinusoidal cells and Kupffer cells (resident macrophages located in the liver) [59, 60].

In addition, elderly patients suffer from the deleterious effects of glycation because their tissues have accumulated glycated products throughout their lives through basal glycation.

## Glycation and oxidation: two related mechanisms that affect diabetic complications and aging

Every step in the glycation process, from the preliminary stages though to the intermediate steps and all the way up the final phase, generates free radicals. Numerous pro-oxidizing molecules are synthesized during glycation, including reactive glyoxal aldehydes, methylglyoxal, and 3-deoxyglucosone. Many glycation products, such as Amadori products and AGEs, subsequently react with oxygen by producing significant quantities of free radicals. The stimulation of RAGE by glycation products can also cause oxidative stress [22]. RAGE is a 45 kDa transmembrane receptor that binds to multiple ligands and belongs to the immunoglobulin superfamily. This receptor is made up of an extracellular domain, a transmembrane domain, and a short cytoplasmic domain that is essential for transduction. It is located in endothelial cells, lymphocytes, monocytes, fibroblasts, polymorphonuclear neutrophils, neurons, smooth muscle cells, pericytes associated with capillaries, and other cells. RAGE is the only AGE receptor able to induce intracellular signal transduction, which, as we will see later, is responsible for various deleterious effects [61].

Also, potentially present in the bloodstream are receptor isoforms that lack transmembrane and intracellular segments. These isoforms are known as the soluble receptor for advanced glycation end products (sRAGE). This term includes all circulating forms of RAGE. There are two types of sRAGE: circulating receptors produced by proteolytic cleavage of a full-length RAGE (catalytic RAGE) and those generated by alternative splicing of the mRNA of a full-length RAGE. sRAGEs circulate in the bloodstream and bind with AGEs, thereby limiting the pathological effect caused when AGEs bind with RAGEs. It appears that these soluble receptors act as decoys by binding to ligands more easily than full-length RAGEs. The use of recombinant sRAGEs in animal trials has been reported to block the development of various pathologies such as cardiovascular diseases, cerebral malfunction, and even cancers [62]. According to some studies, type 2 diabetic patients have lower levels of circulating sRAGE than healthy patients and thus diabetic subjects may be more vulnerable to the damage caused by AGEs.

RAGE can also be activated by other molecules such as:

– the family of S100/calgranulin proteins, which includes pro-inflammatory and pro-oxidizing polypeptides;
– β-amyloid peptides, which are implicated in Alzheimer’s disease and produced through the splicing of the amyloid precursor protein;
– high-mobility group box-1 (HMGB) proteins or amphoterin, a nuclear protein secreted by certain cells when they are stimulated in stressful conditions. Amphoterin is strongly expressed in the central nervous system as it matures and undergoes differentiation. Its binding to RAGEs promotes the growth and invasive quality of glial tumors (brain tumors) [9, 63]. Amphoterin also affects collagen synthesis via the activation of RAGE, resulting in impaired healing [64]

Low levels of RAGE are present in various adult tissues, the exception being lung and skin tissues, where it is highly expressed [65].

The binding of AGE to RAGE has consequences on cellular levels through a series of signaling cascades. During the binding process, transduction first triggers oxidizing mechanisms through the formation of reactive oxygen species followed by the activation of RAS p21 proteins and MAP kinases as well as the transcription factor NF-kB, which activates various genes implicated in inflammation. As a result, the following are expressed in endothelial cells: pro-inflammatory cytokines, interleukins (IL)-1α and IL-6 and tumor necrosis factor-α; chemokines such as monocyte chemoattractant protein-1; and proteins implicated in coagulation, vasoconstriction (endothelin-1), the recruitment of leukocytes, and cellular adhesion (vascular cell adhesion molecule-1). Recruited leukocytes then release cytokines that are RAGE ligands, namely S100/calgranulin and HMGB proteins. As a result, the activation and overexpression of RAGE is self-sustaining, thereby maintaining an inflammatory response that tends to become chronic [8, 66, 67]. The activation of a tissue RAGE (on the surface of neurons, renal podocytes, dermal fibroblasts, *etc*.) also creates the same pro-oxidizing and pro-inflammatory conditions. Thus, glycation and oxidation are two closely related and deleterious mechanisms that sustain each other.

To date, several other AGE-receptors have also been identified consisting of the AGE-receptor complex and some members of the scavenger receptor family [for review, see 68]. In addition, the structural biology of AGE-RAGE complexes is currently being studied to better understand how the different AGEs contribute to RAGE signaling [69].

## The role of glycation in diabetic complications

### Diabetic cardiovascular diseases

Diabetes itself can be an asymptomatic condition. When diet and medical management are insufficient, it gradually and insidiously becomes more serious until symptoms appear.

In the case of diabetes, chronic hyperglycemia stimulates a collection of independent metabolic pathways that lead to vascular malfunction, including glycation, oxidative stress, polyol pathway activation, activation of the protein kinase C pathway, and activation of the hexosamine pathway. In addition to these biochemical pathways, other mechanisms associated with diabetes, including the alteration of platelet function and coagulation properties, also contribute to diabetic cardiovascular abnormalities [66]. Macrovascular lesions are not specific to diabetes, but they are the main cause of diabetes-related mortality for both type 1 and type 2 diabetic subjects. Macroangiopathy refers to damage in large- and medium-sized arteries, including the aorta, coronary arteries, carotid, and arteries in the lower limbs. Medium- and small-sized arteries also experience medial calcification (medial calcinosis), *i.e*. a loss of elasticity in the media and internal elastic lamina of arteries [70, 71]. The serum level of pentosidine is also correlated with arterial thickness and rigidity in subjects with type 2 diabetes [72].

The relative protection of non-menopausal women from coronary heart disease disappears in patients with diabetes. Coronary risk is actually present as soon as the patient develops insulin resistance and before the occurrence of type 2 diabetes. The United Kingdom Prospective Diabetes Study (UKPDS) confirmed that upon the appearance of type 2 diabetes, 10% to 20% of patients are already affected by the clinical complications of artherosclerosis [73, 74]. Chronic hyperglycemia increases the levels of glycated products, which represents a cardiovascular risk factor whose effect is compounded by other risk factors including hypertension, hypercholesterolemia, tobacco and alcohol use, and obesity.

The sub-endothelial extracellular matrix is sensitive to the effects of glycation because it includes long-lived proteins. As mentioned above, extracellular matrix proteins such as fibronectin, laminin, and elastin become rigid when glycated. Increased arterial rigidity leads to higher blood pressure, which results in atherosclerosis and various thrombotic events [75-78]. Another point concerning glycated proteins within the extracellular matrix is that they may trap nitric oxide, a molecule that promotes vasodilation and inhibits various mechanisms that contribute to atherosclerosis. AGE binding to its receptor reduces the activity of endothelial nitric oxide synthase, the enzyme that produces nitric oxide [79]. Endothelial synthesis of prostacyclin, a vasodilator prostaglandin, is also slowed by AGEs whereas AGEs increase the synthesis of endothelin-1, a vasoconstrictor peptide [26, 80-82].

Stimulation of RAGE also triggers a pro-coagulant state due to the reduced activity of thrombomodulin and increased expression of tissue factor [83, 84]. This pro-coagulant state can lead to the formation of a blood clot, which can in turn result in stenosis or potentially thrombosis. Note that diabetic patients are more susceptible to silent myocardial ischemia which is a factor that predicts poor prognosis as it may later lead to major cardiac events [85]. Deterioration in angina can lead to coronary thrombosis, *i.e*. myocardial infarction. Heart attacks are more frequent and more serious among diabetic subjects. The etiology of cardiovascular disease in this group is also very likely to involve iron and iron proteins [86, 87]. Diabetic subjects appear to be at increased risk of stroke, with ten to twenty percent of stroke victims being diabetic. Strokes in diabetic patients are unique in that they are more likely to be ischemic. Prognosis is also less favorable in subjects with chronic hyperglycemia or excessive anxiety, and survivors recover more slowly and remain more disabled than non-diabetic subjects.

Obliterating arteriopathy of the lower limbs is caused by lesions in arteries located in the lower limbs. In diabetic patients, the risk of arteritis in the lower limbs is multiplied by a factor of 4 to 6, and its complications are severe.

Diabetic microangiopathy refers to a collection of complications caused by damage to small blood vessels, mainly capillaries. The retina, glomerulus, and nerves are among the areas most affected. The Diabetes Control and Complications Trial for type 1 diabetes and the UKPDS study for type 2 diabetes demonstrated that aggressive treatment of diabetes can significantly reduce the risk of these complications. Poorly controlled diabetes is often characterized by concurrent damage to the eyes, kidneys, and nerves.

Chronic hyperglycemia first causes hemodynamic changes in the capillary network, including an increase in flow, pressure, and capillary permeability. This condition increases the thrombogenicity of blood by increasing viscosity and platelet aggregation.

### Diabetic retinopathy

On average, diabetic retinopathy occurs around ten years after the onset of the disease. It is the primary cause of blindness in adults living in developed countries. Retinopathy is a common complication of diabetes. Ninety percent of type 1 diabetics and over 60% of type 2 diabetics will suffer from this condition in the first twenty years of the disease’s progression. Twenty percent of type 2 diabetic patients are already affected by retinopathy at the time of their diagnosis with diabetes. Diagnostic methods such as optical coherence tomography, blood glucose levels and blood pressure monitoring, and treatments such as intraocular anti-vascular endothelial growth factor injections make it possible to maintain or improve vision. However, diabetic macular edema remains the primary cause for vision changes [88, 89].

Pathophysiological cascades induced by AGEs play an important role in the progression of diabetic retinopathy and AGEs also play a role in retinal cell death. Proteins in the eye are very sensitive to the formation of AGEs, which accumulate in the retina, vitreous fluid, and lens [90-92]. Once again, AGEs produce a significant number of toxic effects via RAGEs. This type of receptor can be found on the surface of neural cells, endothelial cells, and retinal pigment epithelium cells [93, 94]. When activated, RAGEs appear to play an important role in sustaining inflammation, increasing vascular endothelial growth factor production, neurodegeneration, and microvascular malfunctions associated with diabetic retinopathy [90, 95-98].

### Diabetic nephropathy

Diabetic nephropathy affects 20% to 40% of patients with type 1 and 2 diabetes. In general, this condition follows diabetic retinopathy, and from a clinical viewpoint, progressively affects glomerular filtration [99]. There are several additional risk factors for kidney failure, including hypertension, tobacco use, and obesity. It appears that renal accumulation of AGEs (CML and pentosidine) occurs early in diabetic nephropathy [100-102]. As this pathology develops, an accumulation of proteolysis-resistant extracellular matrix develops within the glomerular basement membrane and mesangium [103-105]. AGEs act through RAGEs to induce transforming growth factor-β release, which plays a major role in deposition of extracellular matrix via the induction of matrix protein synthesis, including collagens (type I, III, and IV) and fibronectin, by renal cells [105,106]. Other pro-fibrotic factors, such as connective tissue growth factors are also synthesized when RAGEs are activated [107]. The increase in the number of fibroblasts and myofibroblasts (activated fibroblasts implicated in tissue repair and fibrosis) as well as the infiltration of macrophages then leads to an excessive accumulation of the extracellular matrix [105]. In addition, it should be noted that the expression of the anti-fibrotic agent, bone morphogenetic protein-7, is decreased in the kidneys of diabetic patients. Once again, as is the case with other diabetic complications, the stimulation of RAGEs plays an important role in diabetic nephropathy [9]. The expression of this receptor is increased with this condition, and its activation results in the apoptosis of podocytes. The autocrine activation of parietal epithelial cells by AGEs thickens the Bowman’s capsule surrounding the glomerulus and potentially alters the efficacy of kidney filtration [108-111]. Glycation products therefore result in renal toxicity, which gradually reduces the kidney filtration capacity and decreases AGEs elimination. As a result, these end products accumulate in the body [112].

### Diabetic neuropathy

Finally, the various types of diabetic neuropathy are characterized by a loss of sensitivity, paresthesia (a sensation of tingling or prickling), and dysesthesia (abnormal sensations triggered by contact or a stimulus) occasionally accompanied by varying degrees of neuropathic pain such as burning or crushing sensations. These problems often lead to a loss of nociception. As a result, they can make injuries or pressure from ill-fitting footware imperceptible to patients, which combined with healing deficit can result in a pathology known as "diabetic foot". This condition is very difficult to heal and often leads to amputation. The loss of peripheral sensitivity can also lead to an unstable posture and increase the number of falls suffered by elderly diabetic patients. These peripheral neuropathies can be aggravated by orthostatic hypotension, urogenital disorders, and silent myocardial ischemia [113-121]. The capillaries that perfuse nerves are also affected by AGEs. Indeed, extracellular glycation thickens the basement membrane and increases parietal permeability by changing its electrical charge. The activation of endothelial RAGEs in perineural and endoneurial blood vessels sets the stage for vascular malfunction. All of these processes associated with glycation lead to circulatory issues in the capillaries and the onset of hypoxia, which affects nervous tissue. They also weaken the immune system, which could increase the likelihood of skin wounds and infections in the feet [122, 123].

## Endogenous glycation and aging

Aging is an overall change in a collection of physiological functions and an increased susceptibility to various diseases. Aging is also associated with chronic, low-level inflammation evidenced by increased blood levels of inflammatory mediators that contribute to functional disruptions, the development of chronic disease, and the state of fragility that occurs throughout aging [124-127].

Moreover, the accumulation of AGEs has been found in many parts of the body, including the blood, blood vessel walls, retina, lens, kidney, brain, peripheral nerves, joints, and skin. The build-up of these products results in significant changes in the metabolism, appearance, and biomechanical properties of these organs [21, 128, 129].

AGEs accumulate over time because kidney function decreases with age regardless of the subject having diabetes. However, aging itself is a condition that favors AGE formation and accumulation due to the age-associated increase in oxidative stress [125]. In addition, repair processes are less efficient. Basal glycation that occurs over a number of years contributes to aging and can lead to various pathologies by exerting deleterious effects that, while similar to those caused by diabetes, are expressed later and often to a lesser degree [130]. In contrast, it can also be hypothesized that the dietary restriction and qualitative and quantitative changes observed in the elderly diet, may limit their consumption of exogenous AGEs [131].

The accumulation of AGEs during aging is especially notable in structures that contain collagen. A build-up of glycation products is correlated with increased rigidity in the arteries, tendons, and skin [19]. AGEs play adverse proinflammatory roles in osteoporosis [132] and the serum level of sRAGE could therefore have a potential diagnostic role in the monitoring of osteoporosis progression [133].

AGEs also play a role in the aging of skeletal muscle. Muscle mass and strength decrease during the aging process, which can increase the fragility and dependence of the elderly [124]. Glycation and oxidation, especially with respect to lipids, also affect the pathophysiological process of age-related macular degeneration and formation of cataracts, thereby disrupting the quality of vision and the visual field [134-138].

### Glycation and skin aging

In aging, the skin becomes dryer, thinner, and less elastic and dark spots and wrinkles also appear. The skin’s sensory abilities also change. Undoubtedly, skin aging is strongly influenced by exterior factors such as tobacco use, exposure to ultra-violet (UV) rays, pollution, and lifestyle [139].

The skin of elderly patients is characterized by a thinner epidermis, a lack of dermal papillae, and an atrophied and disorganized dermal extracellular matrix (including the presence of fragmented collagen bundles and thickened, denatured elastic fibers). UV rays accelerate skin aging through a variety of mechanisms (induction of oxidative stress, increased production of metalloproteases, *etc*.). Aged skin that is exposed to the sun is characterized by a more significant accumulation of denatured elastic fibers (solar elastosis).

**Figure 3.**
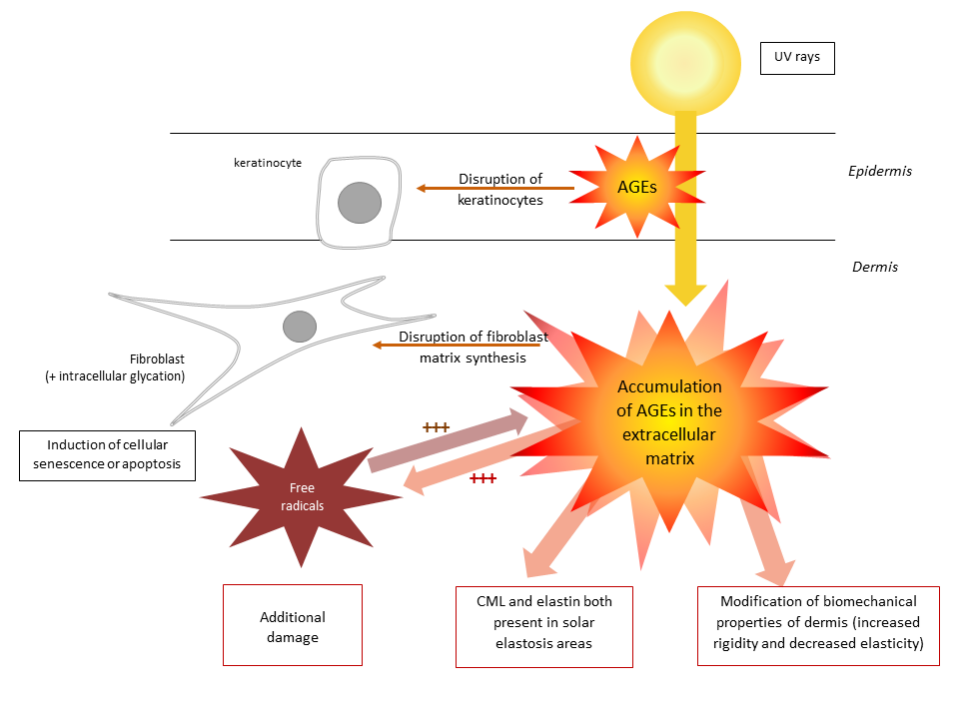
Diagram of the effects (suggested by various studies) of glycation on skin aging.

Glycation is a process that contributes to skin aging in a variety of ways (see Table 1 and Fig. 3).

**Table 1:** Summary of effects (suggested by various studies) of glycation on skin aging.

**Glycation within the dermal and epidermal extracellular matrix**
➢ Increased rigidity of the skin and decreased elasticity
➢ Activation of RAGE: secretion of cytokines and growth factors
➢ Induction of senescence and apoptosis in fibroblasts and disruption of keratinocytes
➢ Changes in the synthesis of components of the extracellular matrix and of metalloproteinases
**Intracellular glycation**
➢ Decreased effectiveness of the proteasome when its enzymes are affected by glycation
➢ Accumulation of glycated vimentin within fibroblasts and decreased contractile activity of these cells in collagen gels
**Effects of UV rays**
➢ Effect of UVA rays on certain AGEs in the skin: stimulation of the products of reactive oxygen species, which are not eliminated as effectively following the glycation of catalase and superoxide dismutase
➢ Stimulation of the production of AGEs and of the expression of RAGEs in the skin due to exposure to sunlight

AGEs accumulate with age in the extracellular matrix of the dermis and epidermis. They bind with long-lived components of the extracellular matrix like collagen and elastin fibers and play a role in intermolecular connections. Glycation modifies the skin’s physical properties, rendering it more rigid and less elastic. It is important to note that RAGEs are concentrated in the skin, especially on keratinocytes, fibroblasts, and dendritic cells. RAGE activation contributes to changes in cellular activity and the production of cytokines and growth factors [140-142].

An *in vitro* study has demonstrated that AGEs in the epidermis could disrupt the migration and proliferation of keratinocytes, thereby resulting in a decreased ability for the skin to repair itself and impaired wound healing [143]. In addition, the accumulation of AGEs could have a direct or indirect effect on skin pigmentation and its optical qualities [144, 145].

*In vitro* studies have shown that CML, when bound to skin collagen, stimulates apoptosis in human fibroblasts through the activation of RAGE [146]. AGEs could therefore promote the processes of cellular senescence and apoptosis, which would contribute to the loss of cells that is observed in skin aging [35, 147, 148].

AGEs also appear to modify the equilibrium and stability of the skin by changing the synthesis of molecules in the extracellular matrix and affecting the synthesis and activity of metalloproteinases (MMPs) [149]. An *in vitro* study using a three-dimensional model of reconstructed skin, with a dermal section in which the collagen had been modified by glycation, demonstrated a number of changes including perturbations of MMP production, an increase in type IV collagen and laminin in the basement membrane zone and expansion of alpha 6 and beta 1 integrins in suprabasal layers of the epidermis [150]. However, these modifications need be confirmed in *in vivo* skin aging.

Intracellular molecules are also affected by glycation. Glycation reduces the activities of proteasomal enzymes *in vitro*. Glycation also appears to hinder the destruction of abnormal proteins. This form of glycation also seems to decrease the contractile properties of fibroblasts/myofibroblasts in three-dimensional collagen gels, suggesting an alteration in cell-matrix interactions [35, 151].

**Figure 4.**
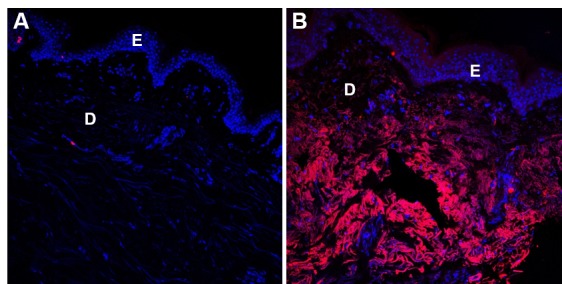
Carboxymethyl-lysine immunostaining (red) on normal human skin: glycated (A) and non-glycated (B) areas Nuclei are stained using DAPI (blue). E: epidermis; D: dermis. Magnification x200.

UVA rays lead to the formation of free radicals through the creation of glycated products (pentosidine) and a variety of other complex mechanisms. Pentosidine produces harmful free radicals [152, 153]. Glycation also seems to decrease the activity of naturally protective enzymes such as catalase and superoxide dismutase [154]. As a result, the body’s natural defense systems against reactive oxygen species are weakened, resulting in the appearance of the signs of aging. CML (Fig. 4) and modified elastin can both be found in areas affected by solar elastosis, suggesting that AGEs may in part be responsible for solar elastosis [155, 156].

### Glycation and cerebral aging

Alzheimer’s disease is a neurodegenerative disease, characterized by neuronal death that most often affects people over the age of sixty-five. It manifests as a progressive loss of memory, spatial and temporal orientation, and reasoning leading to a concomitant and considerable reduction in the patient’s autonomy. Alzheimer’s disease is the result of two pathological processes that first develop in the hippocampus and then spread to the cerebral cortex: 1) the aggregation of hyper-phosphorylated tau proteins in neurons and 2) the accumulation of β-amyloid peptides on their surface [157, 158]. Current treatments have only a moderate effect on symptoms and progression of the disease [159, 160].

Studies have shown that diabetes increases a patient’s risk of developing Alzheimer’s disease. The Rotterdam study examined over 6,000 patients without any signs of dementia, around 11% of whom were diagnosed with diabetes mellitus. Subjects were then monitored for one year. The study showed that diabetes nearly doubled a patient’s risk of developing dementia and Alzheimer’s disease. Diabetic subjects being treated with insulin were the most at risk. The Honolulu-Asia Aging Study demonstrated that diabetic subjects had a significantly higher risk of developing Alzheimer’s disease, vascular dementia, and memory loss [161-163]. Nevertheless, other studies have reached different conclusions. A Canadian study did not find any correlation between diabetes and the incidence of any type of dementia. It did however estimate that suffering from diabetes doubled a patient’s risk of developing vascular cognitive impairment [164-166]. Moreover, a post-mortem histological study of Alzheimer’s patients did not find a greater density of characteristic protein deposits in diabetic subjects [167].

While the role of glycation does not appear to be "clear cut", studies using immunodetection found pentosidine and pyrraline in amyloid plaque and neurofibrillary deposits. However, these studies did not irrefutably prove these products are biomarkers for the disease [169-170]. Indeed, AGEs derived from glyceraldehyde have been primarily found in the cytosol of neurons, for example in the Hirano bodies (cytoplasmic inclusions that accumulate in Alzheimer’s disease), especially in the hippocampus and parahippocampal gyrus. In addition, glycation products have also been found in patients with other neurodegenerative diseases such as Pick’s disease and Lou Gehrig’s disease [170-172]. Not only are glycated products found in the very same lesions caused by Alzheimer’s disease, it has also been shown that AGEs and RAGEs play a role in neurotoxic mechanisms.

**Figure 5.**
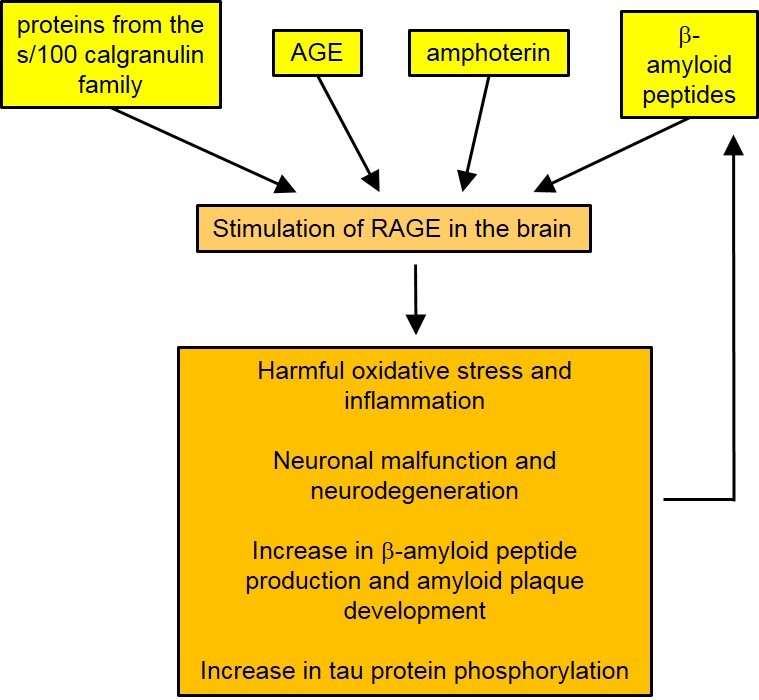
The role played by stimulated RAGEs in Alzheimer’s disease as suggested by various studies.

In the brain, RAGEs are expressed on the surface of neurons, astrocytes, microglia, and vascular cells. In areas of the brain affected by the disease, such as the hippocampus, the expression of this receptor increases. The accumulation of AGEs and β-amyloid peptides in amyloid plaque induces the overexpression of RAGE in the brains of Alzheimer’s patients [173, 174]. The significant number of activated glial cells and increased synthesis of inflammatory mediators appears to trigger a state of oxidative stress and chronic inflammation in the brain [175-177]. The activation of RAGE can also alter synaptic transmission and decrease neuronal plasticity. It also causes mitochondrial dysfunction, which then contributes to neuronal degeneration [174, 178, 179]. RAGE also appears to be implicated in the appearance of primary lesions in the disease [180-183] (Fig. 5).

RAGE acts as a blood-brain barrier transporter by increasing the flow of β-amyloid peptides to the brain. Its movement from the brain to blood is mediated by the LDL receptor-related protein-1 [178, 184]. The expression of RAGE in the blood-brain barrier is higher in Alzheimer’s patients [185, 186]. The accumulation of amyloid plaques in the brain, in the case of Alzheimer’s disease, could therefore be a result of insufficient removal of β-amyloid peptides through the blood brain barrier. sRAGEs could be implicated in this pathology. Note that these sRAGEs bind to AGEs in the bloodstream, thereby inhibiting the stimulation of RAGEs and the resulting toxic effects from this process. Moreover, sRAGEs help to remove β-amyloid peptides from the brain via the blood brain barrier. An Italian team reported that average levels of sRAGEs were significantly lower in Alzheimer’s patients. It is therefore possible that a low concentration of receptors in the bloodstream increases brain cell vulnerability to the toxic effects of amyloid plaques. However, it remains to be determined whether a low level of sRAGEs is a cause or a consequence of Alzheimer’s disease [184-188].

The discovery of AGEs in the brains of non-diabetic Alzheimer’s patients supports the hypothesis of a local deregulation of glucose use by neurons. Specifically, this phenomenon is unrelated to the chronic hyperglycemia seen in type 1 or type 2 diabetes. Recent studies on Alzheimer’s disease report defects in the expression of insulin, its receptors, and insulin-like growth factor. Abnormal insulin signaling can also be noted. This suggests that Alzheimer’s disease is therefore the result of localized insulin resistance leading some scientists to refer to it as type 3 diabetes. As a result, we can hypothesize that glycation occurs in a context of localized cerebral hyperglycemia that is potentially caused by reduced insulin sensitivity. However, there is no proof that the formation of AGEs does not occur later in the disease’s pathophysiology [189-191]. It remains unclear whether the glycation of proteins causes amyloid plaques to form and neurofibrillary degeneration to occur, or if it is consequence of their occurrence. In addition, evidence has recently emerged suggesting a contributing role of AGEs in the pathology of multiple sclerosis [192].

## Conclusion

Glycation is a common mechanism found in many disorders and molecular precursors, particularly the reactive dicarbonyl metabolite methylglyoxal, are key in the development and accumulation of damage. AGEs are recognized by various cells in organs through their corresponding receptor, RAGE, and have been linked to various diseases [193]. Indeed, glycation damage is of prime importance since it has been shown to be involved in nephropathy, diabetes and many other disorders. Oxidative stress, which appears even at early stages of the reaction and at physiological glucose concentrations, plays a critical role. Over the last decade, different therapeutic strategies have been developed, targeting the formation and degradation of AGEs, as well as the interaction of these AGEs with their receptors [194]. For example, it has been shown recently that the activation of the nuclear factor erythroid 2-related factor 2 (Nrf2)/Kelch-like ECH-associated protein 1 (Keap1) pathway represents an interesting defense response, through regulation of glutathione level, against the formation of methyglyoxal-modified proteins which is accepted as evidence of carbonyl stress [195]. In addition, under physiological circumstances, methylglyoxal is detoxified by the glyoxalase system, glyoxalase I being the key enzyme in the anti-glycation defense. Bioactive inducers of this enzyme represent new treatment strategies for age-related disorders where methylglyoxal is implicated [196, 197]. New approaches such as redox proteomics appear to have great potential for detecting target proteins. Finally, potential pharmacological approaches to circumvent the deleterious effects of AGEs by reducing exogenous and endogenous sources of AGEs, increasing the breakdown of existing AGEs, or inhibiting AGE-induced inflammation, including the use of plant products with anti-glycation activity, are also promising [198, 199]. Clearly, dietary consumption has recently been identified as a major environmental source of pro-inflammatory AGEs in humans and studies suggest the need to limit the dietary sources of AGEs, including added sugars, to prevent the development of oxidative/carbonyl stress, metabolic diseases and related comorbidities in the aged and vulnerable population [200]. Finally, the detection of fluorescent AGEs within the skin by non-invasively measuring skin autofluorescence currently remains an interesting tool to evaluate the contribution of AGEs to pathological disease processes [201].
